# Systematic functional perturbations uncover a prognostic genetic network driving human breast cancer

**DOI:** 10.18632/oncotarget.16244

**Published:** 2017-03-15

**Authors:** Tristan Gallenne, Kenneth N. Ross, Nils L. Visser, Salony, Christophe J. Desmet, Ben S. Wittner, Lodewyk F.A. Wessels, Sridhar Ramaswamy, Daniel S. Peeper

**Affiliations:** ^1^ Department of Molecular Oncology, The Netherlands Cancer Institute, Plesmanlaan, CX, Amsterdam, The Netherlands; ^2^ Department of Molecular Carcinogenesis, The Netherlands Cancer Institute, Plesmanlaan, CX, Amsterdam, The Netherlands; ^3^ Faculty of EEMCS Delft University of Technology, Delft, The Netherlands; ^4^ Massachusetts General Hospital Cancer Center, Boston, MA, USA; ^5^ Harvard Medical School, Boston, MA, USA; ^6^ Broad Institute of Harvard & MIT, Cambridge, MA, USA; ^7^ Harvard Stem Cell Institute, Cambridge, MA, USA; ^8^ Harvard-Ludwig Center for Cancer Research, Boston, MA, USA; ^9^ Current address: Merus B.V., Padualaan, CH Utrecht, The Netherlands

**Keywords:** breast cancer, metastasis, prognosis, tumor biology

## Abstract

Prognostic classifiers conceivably comprise biomarker genes that functionally contribute to the oncogenic and metastatic properties of cancer, but this has not been investigated systematically. The transcription factor Fra-1 not only has an essential role in breast cancer, but also drives the expression of a highly prognostic gene set. Here, we systematically perturbed the function of 31 individual Fra-1-dependent poor-prognosis genes and examined their impact on breast cancer growth *in vivo*. We find that stable shRNA depletion of each of nine individual signature genes strongly inhibits breast cancer growth and aggressiveness. Several factors within this nine-gene set regulate each others expression, suggesting that together they form a network. The nine-gene set is regulated by estrogen, ERBB2 and EGF signaling, all established breast cancer factors. We also uncover three transcription factors, MYC, E2F1 and TP53, which act alongside Fra-1 at the core of this network. ChIP-Seq analysis reveals that a substantial number of genes are bound, and regulated, by all four transcription factors. The nine-gene set retains significant prognostic power and includes several potential therapeutic targets, including the bifunctional enzyme PAICS, which catalyzes purine biosynthesis. Depletion of PAICS largely cancelled breast cancer expansion, exemplifying a prognostic gene with breast cancer activity. Our data uncover a core genetic and prognostic network driving human breast cancer. We propose that pharmacological inhibition of components within this network, such as PAICS, may be used in conjunction with the Fra-1 prognostic classifier towards personalized management of poor prognosis breast cancer.

## INTRODUCTION

Gene-expression patterns of primary breast cancers aid clinicians in predicting the risk of metastatic disease [1–6]. Some prognostic signatures have recently been prospectively validated, highlighting their clinical value [7, 8]. Such classifiers conceivably comprise biomarker genes that, in fact, functionally contribute to the oncogenic and metastatic properties of the tumors, but this has not been investigated systematically. We recently reported that the transcription factor Fra-1 (Fos-related antigen-1, a component of AP-1 transcription-regulating complexes) is a key promoter of breast cancer cell metastasis [9]. Subsequent work suggested a role for Fra-1 in breast cancer stem cells [10]. We also showed that the Fra-1 transcriptome is endowed with high prognostic power for clinical outcome of breast cancer patients [9]. It has been suggested that in a data-driven approach, targets acting downstream of a transcription factor, rather than the transcription factor itself, possess better distinguishing features, because they reflect the activity of the transcription factor [11]. Therefore, we hypothesized that, in addition to its prognostic value, the Fra-1 dependent transcriptome may harbor one or more genes that drive breast cancer.

## RESULTS

**Figure 1:**
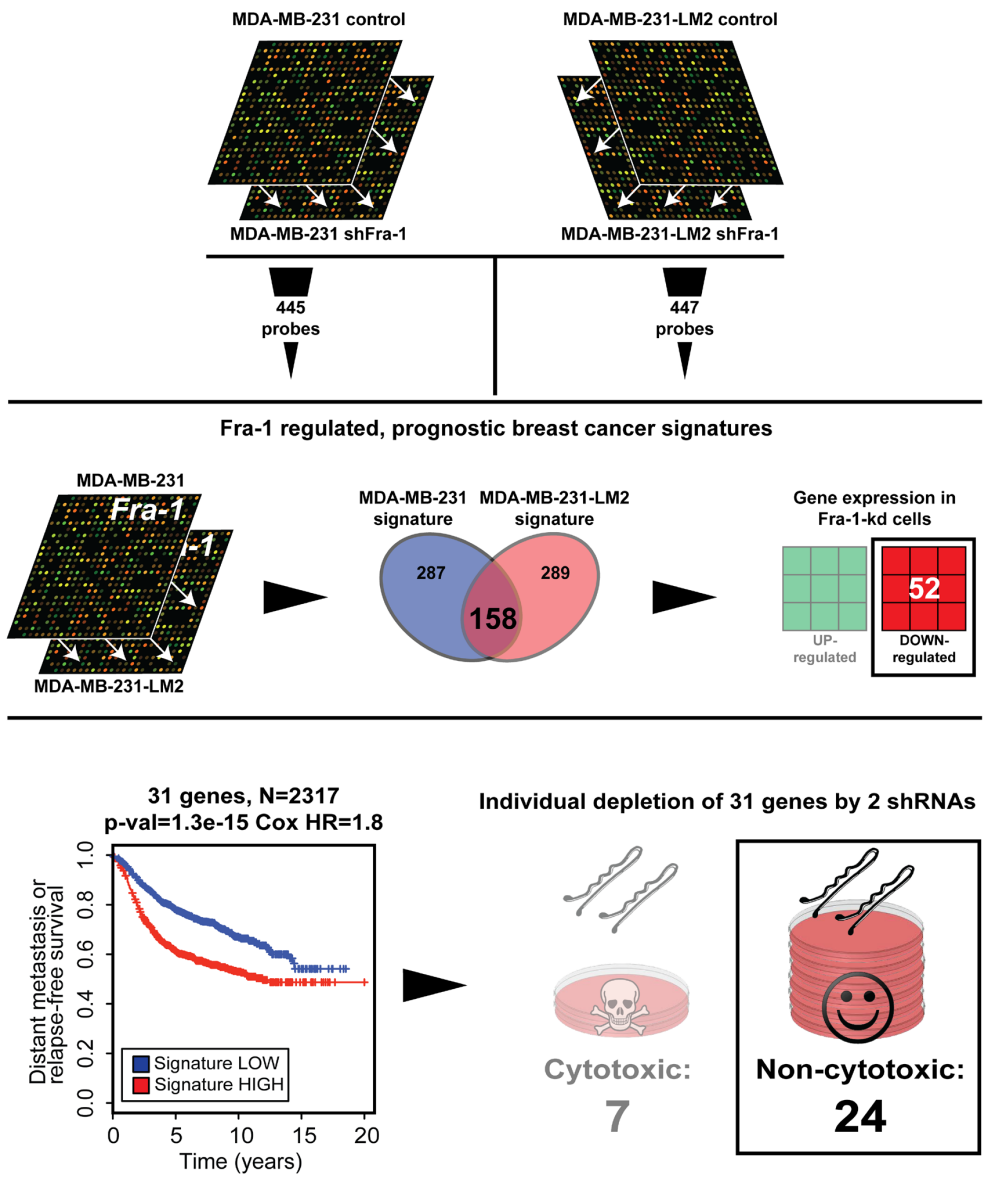
Identification of a Fra-1-dependent prognostic gene set Outline of the procedure used to generate the 24-gene set common between the MDA-MB-231 and LM2 Fra-1-dependent signatures, which is down-regulated by the Fra-1 shRNAs, and highly expressed in poor prognosis patients. Also shown is in a Kaplan-Meier curve for 31-gene set-high samples and 31-gene set-low samples for time to distant metastasis (if available) or relapse with one-sided log-rank p-value and Cox proportional hazards model hazard ratio between the 31-gene set-high and 31-gene set-low groups (see Methods). Only sRNAs that were non-cytotoxic *in vitro* were selected for *in vivo* study..

To investigate this, we performed a systematic functional perturbation of Fra-1 signature genes. First, we compared the gene-expression profiles of control and Fra-1-depleted MDA-MB-231 cells, a triple-negative basal breast cancer cell line, and of its highly metastatic derivative LM2 cells [12]. Among the probes that were significantly regulated by two independent shRNAs targeting Fra-1 (*P* < 1×10^−6^) in both cell lines, we selected those showing a prognostic value in a cohort of 509 breast cancer patients. We subsequently generated gene-expression signatures from both cell lines, comprising 445 and 447 probes respectively (Figure 1, upper panel; see Methods). Among the 158 genes common between the Fra-1 signatures in the two cell lines, we selected those that were downregulated by both Fra-1 shRNAs. This yielded 52 genes (Figure 1, middle panel), from which we selected those that were highly expressed specifically in poor prognosis breast cancer patients. This selection produced a set of 31 genes (Table 1) that were expressed at higher levels than the median in the poor prognosis patients group, and lower than the median in the good prognosis patients group. Using 2317 human breast cancer gene-expression profiles encompassing publicly available breast cancer datasets, we determined that expression of this 31-gene set significantly correlates with clinical outcome of breast cancer patients (Figure 1, bottom panel).

**Table 1:** The 31-gene Fra-1-dependent signature

Gene symbol	Description
ABHD11	abhydrolase domain containing 11
ADORA2B	adenosine A2b receptor
AURKB	aurora kinase B
BIRC5	baculoviral IAP repeat-containing 5 (survivin)
CENPM	centromere protein M
CHAF1A	chromatin assembly factor 1, subunit A (p150)
CHML	choroideremia-like (Rab escort protein 2)
E2F1	E2F transcription factor 1
EZH2	enhancer of zeste homolog 2
FEN1	flap structure-specific endonuclease 1
FOXM1	forkhead box M1
H2AFZ	H2A histone family, member Z
IGFBP3	insulin-like growth factor binding protein 3
MCM10	MCM10 minichromosome maintenance deficient 10
MCM2	MCM2 minichromosome maintenance deficient 2, mitotin
MTDH	metadherin
PAICS	phosphoribosylaminoimidazole carboxylase,
	phosphoribosylaminoimidazole succinocarboxamide synthetase
PCOLN3	procollagen (type III) N-endopeptidase
PHLDA1	pleckstrin homology-like domain, family A, member 1
PPP2R3A	protein phosphatase 2 (formerly 2A), regulatory subunit B'', alpha
PTGES	prostaglandin E synthase
PTP4A1	protein tyrosine phosphatase type IVA, member 1
RRP1	ribosomal RNA processing 1 homolog (S. cerevisiae)
SCD	stearoyl-CoA desaturase (delta-9-desaturase)
SEC14L1	SEC14-like 1
SFN	stratifin
SH3GL1	SH3-domain GRB2-like 1
SMTN	smoothelin
TJAP1	tight junction associated protein 1 (peripheral)
TRFP	Trf (TATA binding protein-related factor)-proximal homolog
YTHDF1	YTH domain family, member 1

Next, we investigated the individual contribution of these poor prognosis genes to outgrowth and metastasis of human breast cancer cells. We systematically depleted each of the 31 genes in LM2 cells using lentiviral transduction of shRNAs (Figure 1, bottom panel). Silencing of seven of these genes (*AURKB, FOXM1, MCM2, MCM10, PCOLN3, SCD* and *SMTN*) had a strong cytotoxic or cytostatic effect *in vitro*. Although possibly of interest, we decided not to pursue these genes in *in vivo* analyses to avoid confounding straight lethal effects.

**Figure 2:**
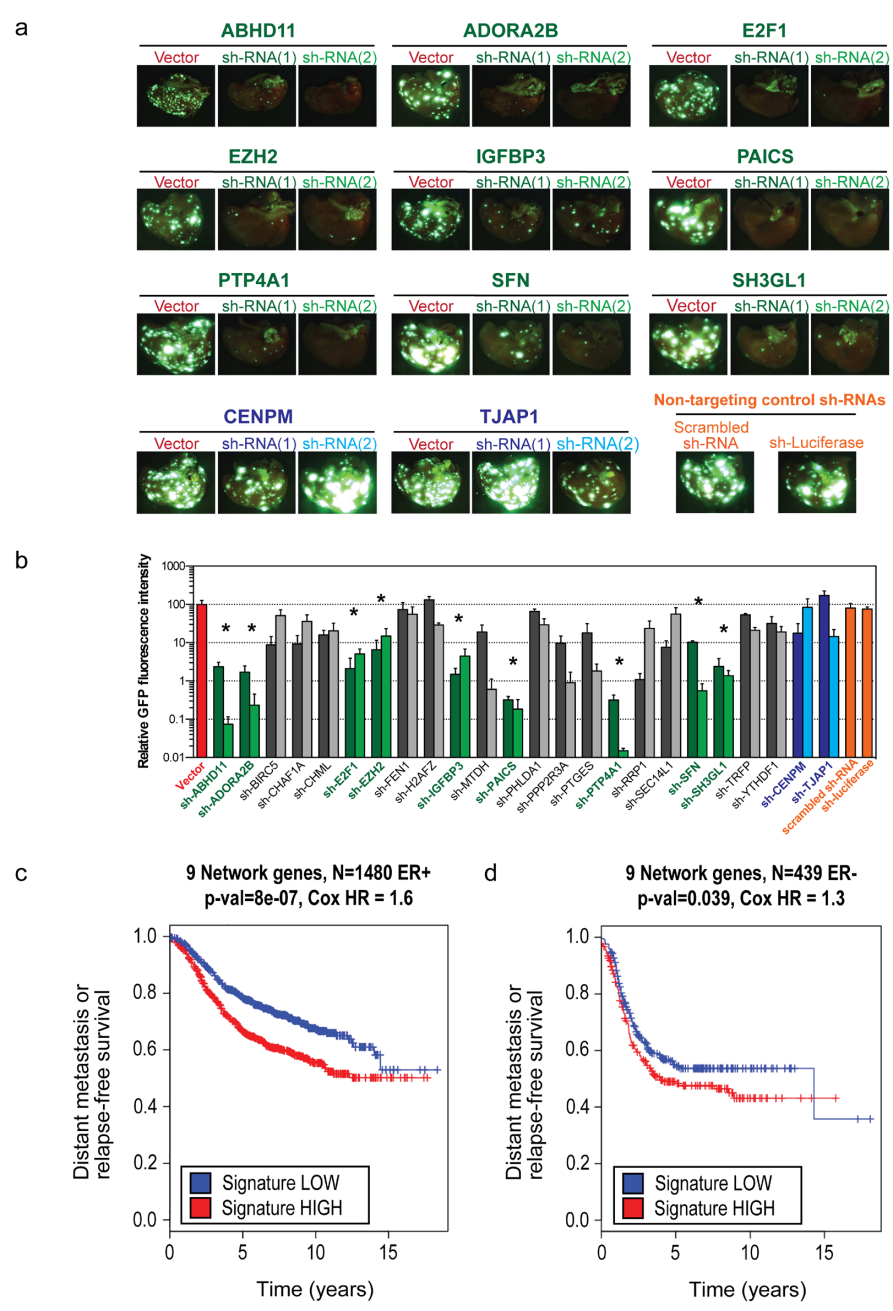
Nine genes validate *in vivo* and retain prognostic power in both ER+ and ER- breast cancer subtypes **a**. Representative fluorescence imaging of the lungs of mice inoculated intravenously with 10^5^ GFP-labeled LM2 cells expressing a control vector or two independent shRNAs directed against the nine genes, 5 weeks after inoculation. Also shown are representative pictures of genes from the set of 24 that did not validate (in blue) **b**. Quantification of the GFP fluorescence in a. (*n* = 3 lungs, error bars: S.E. *p < 0.05 following a one-way ANOVA test). **c**. Kaplan-Meier curve for 9-gene set-high samples and 9-gene set-low samples for time to distant metastasis (if available) or relapse in a sub group of ER+ breast cancer patients (*n* = 1480). One-sided log-rank p-value and Cox proportional hazards model hazard ratio between the 9-gene set-high and 9-gene set-low groups (see Methods) are also shown. **d**. Same as for c, but in a subgroup of ER- breast cancer patients (*n* = 439).

Successful knockdowns for all 24 remaining poor prognosis genes were confirmed prior to *in vivo* inoculation (Supplementary Figure 1). GFP-labeled LM2 cells expressing either one of several controls or one of two independent shRNAs directed against each of the remaining 24 genes were inoculated intravenously into immunocompromised mice. Five weeks later, mice were sacrificed and pulmonary colonization was quantified by fluorescence imaging (Figure 2a). Because of the considerable number of genes in the Fra-1 classifier, we expected that the contribution of single genes would be limited. In contrast, whereas 15 genes had no, or only a moderate inhibitory effect, we identified nine genes whose depletion strongly inhibited experimental metastasis, *ABHD11*, *ADORA2B*, *E2F1*, *EZH2*, *IGFBP3*, *PAICS*, *PTP4A1*, *SFN* and *SH3GL1*. Their contribution to metastasis ranged from one (e.g., *EZH2*) to three or four logs (e.g., *PTP4A1* and *PAICS*; Figure 2b). Consistent with this, for ADORA2B (encoding the Adenosine receptor A2B), we have previously shown that its inhibition, either genetically or pharmacologically, strongly impairs lung colonization of breast cancer cells [9]. Notably, these nine genes retained prognostic power both in ER+ and ER- breast cancer patients (Figure 2c, 2d).

**Figure 3:**
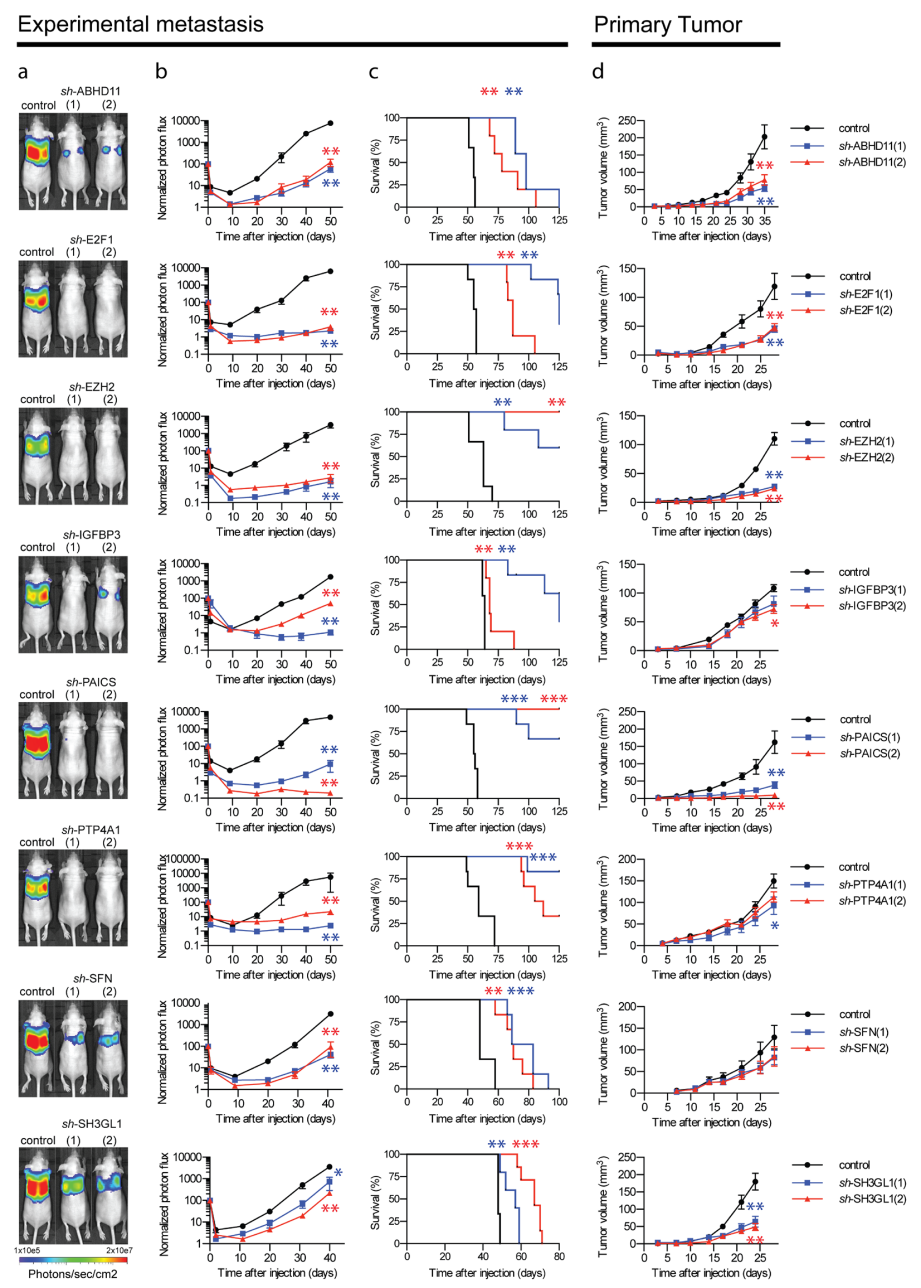
Essential contribution of nine individual genes to primary and metastatic breast tumor growth. Representative bioluminescence images **a**. and quantification of the luminescence signal as a function of time in the lungs of mice **b**. injected intravenously with LM2 cells expressing a control vector or two independent shRNAs directed against the indicated genes (*n* = 6 mice, error bars: S.E.M., *p < 0.05, **p < 0.01, following a Mann-Whitney U-test). **c**. Kaplan-Meier curves for survival of the mice injected intravenously with LM2 cells (2×10^5^ cells) expressing a control vector or two independent shRNAs directed against the indicated genes (*n* = 6 mice, **p < 0.005, ***p < 0.001 following a Mantle-Cox Logrank test). Mice were euthanized when clinical symptoms became apparent. **d**. *In vivo* growth curve of primary tumors formed by LM2 cells expressing a control vector or one of two independent shRNAs directed against the indicated genes, injected in the 4^th^ mammary fat pad on both flanks (*n* = 6 tumors, error bars: S.E.M, *p < 0.05, **p < 0.01, following a Mann-Whitney U-test). Experiments were terminated when the number of animals sacrificed due to tumor burden in one or more experimental groups reached half of the original starting size.

We validated the activity of each of these genes in an independent experiment using non-invasive *in vivo* bioluminescence imaging. This confirmed that the Fra-1 poor prognosis signature harbors nine genes, each of which is critically required for the development of pulmonary cancer colonization (Figure 3a, 3b). Importantly, individual depletion of all of the poor prognosis genes significantly prolonged survival of recipient mice (Figure 3c).

To examine any contribution of these genes not only to experimental metastasis, but also to primary breast cancer growth, we inoculated stably depleted cells orthotopically into the mammary fat pad of immunocompromised mice. This demonstrated that several factors encoded by the poor prognosis gene set critically contributed to the expansion of primary tumors, particularly PAICS and EZH2 (Figure 3d). This was associated with the requirement for each of these genes to allow breast cancer cells to form colonies in semi-solid medium, an *in vitro* hallmark of oncogenic activity (Supplementary Figure 2). This was shown also for three additional human breast cancer cell lines, excluding a cell type-specific effect. The growth-inhibitory effects were generally more pronounced *in vivo* than *in vitro* 2D proliferation (Supplementary Figure 3a).

To determine whether the observed growth-inhibitory effects were not solely attributable to proliferation effects, we examined the correlation of meta-gene from the nine-gene signature with a proliferation score in the TCGA breast cancer data (Supplementary Figure 3b for all breast cancers; Supplementary Figure 3c for the TNBC subset). These results showed a moderate association between the nine-gene meta-gene and proliferation as one would expect, although not enough to explain the entirety of growth inhibition. The expression of *ABHD11, ADORA2B, E2F1, EZH2, PAICS, SFN* and *SH3GL1* was significantly higher in grade III than in grade I breast tumors (Supplementary Figure 4). Thus, the nine-gene set is not only prognostic but also causally linked to the ability of breast cancer cells to form primary tumors and metastases in mice.

**Figure 4:**
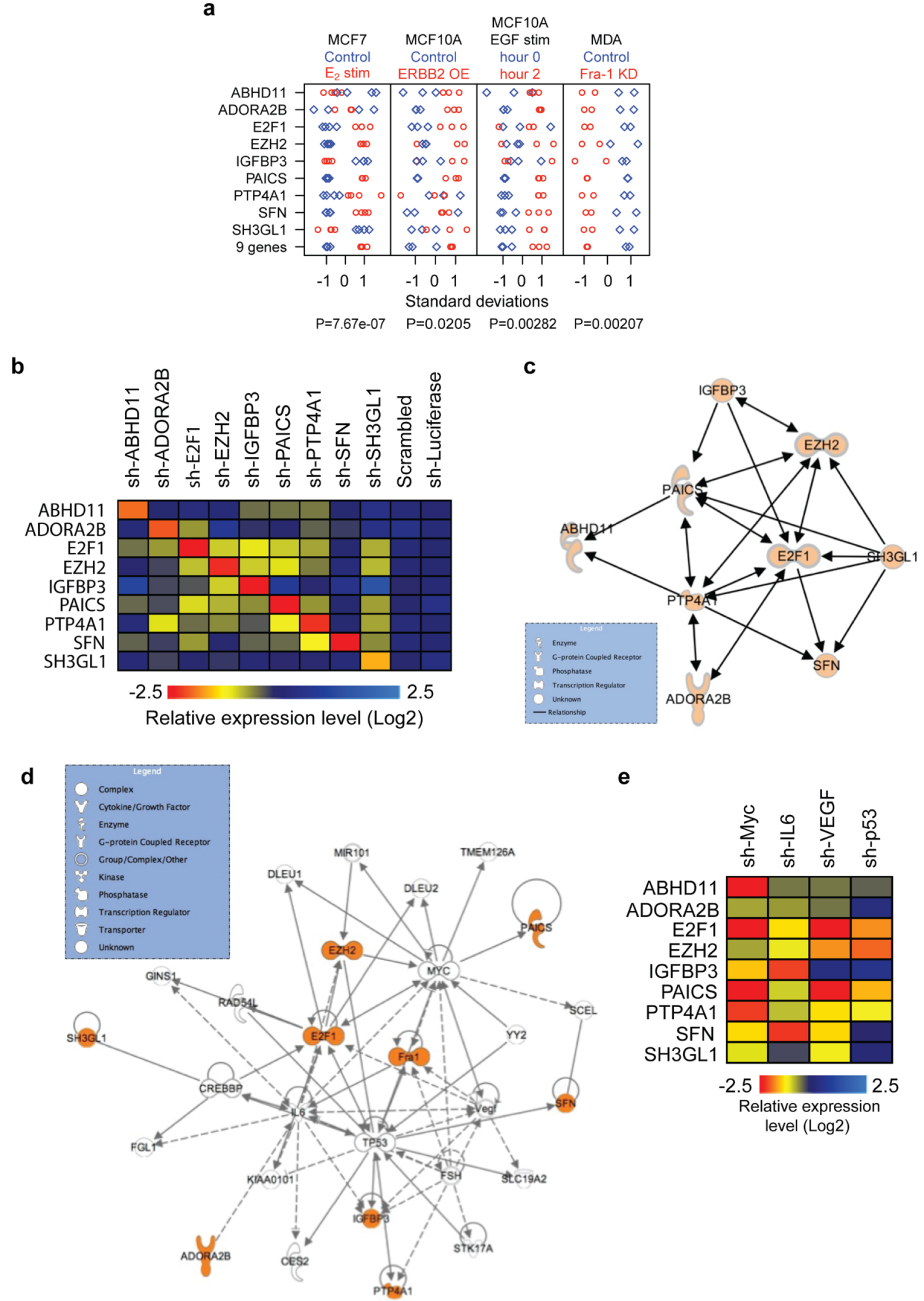
A genetic network driving breast cancer **a**. Nine different genes and a nine-gene metagene (created by averaging all nine genes) correlated with different experimental manipulations, as previously described [9, 16]. Plots displaying genes / metagene in standard deviation units after 17β-estradiol stimulation (red circles) *versus* control (blue diamonds) in MCF7 cells; ERBB2 overexpression (red circles) *versus* control (blue diamonds) in MCF10A cells; EGF stimulation (red circles) *versus* control (blue diamonds) in MCF10A cells; and Fra-1 knockdown (red circles) *versus* control, scrambled hairpin (blue diamonds) in MDA-MB-231 cells. Circles and diamonds represent independent replicates. **b**. Heat map showing the relative expression levels of the components of the nine-gene set in LM2 cells expressing two independent shRNAs directed against the indicated genes. Expression values from the two independent shRNAs were averaged. **c**. Network illustrating the functional connections from b, based on statistically significant regulations with two independent shRNAs (see Supplementary Figure 5a). **d**. Network of components of the nine-gene set, Fra-1 and other associated genes identified using the IPA algorithm (www.ingenuity.com). The nine-gene set and Fra-1 are highlighted in orange. **e**. Heat map showing the relative expression levels of components of the nine-gene set in LM2 cells expressing two independent shRNAs directed against MYC, IL-6, VEGF or TP53, as indicated. Expression values from the 2 independent shRNAs were averaged, measured by quantitative RT-PCR (See Supplementary Figure 5b).

Since genes from prognostic gene-expression signatures may interconnect [13, 14], we next considered the possibility that the proteins encoded by the nine-gene set, which have seemingly unrelated functions, do in fact communicate with one another. Supporting such a model was our observation that the average expression of these 9 genes considered as a set (i.e., a meta-gene) was significantly regulated by estrogen stimulation in ER-positive MCF7 breast cancer cells and by ERBB2 overexpression or EGF stimulation in MCF10A breast epithelial cells (Figure 4a). Thus, components of the nine-gene set are regulated by estrogen, ERBB2 and EGF signaling. Therefore, we examined whether individual factors in this 9-gene set influence each other's expression. Indeed, individual depletion of 7/9 genes strongly affected the expression of several of the other genes (Figure 4b; Supplementary Figure 5a). This mutual expression dependency could be captured in a hypothetical connectivity map based on IPA analysis (www.ingenuity.com) (Figure 4c).

Expanded IPA analysis further suggested the presence of additional network factors (Figure 4d), including several proteins we and others have previously associated with (breast) cancer, particularly (mutant) TP53, MYC, IL-6 and VEGF [15–17]. To functionally validate these computational predictions, we depleted these four genes individually and determined the expression of the nine genes. The absence of each of these genes strongly suppressed most other network genes (Figure 4e, Supplementary Figure 5b). These results raised the possibility that the nine genes might be part of a broader genetic network that is regulated by four transcription factors (TFs): Fra-1, MYC, TP53 and E2F1.

**Figure 5:**
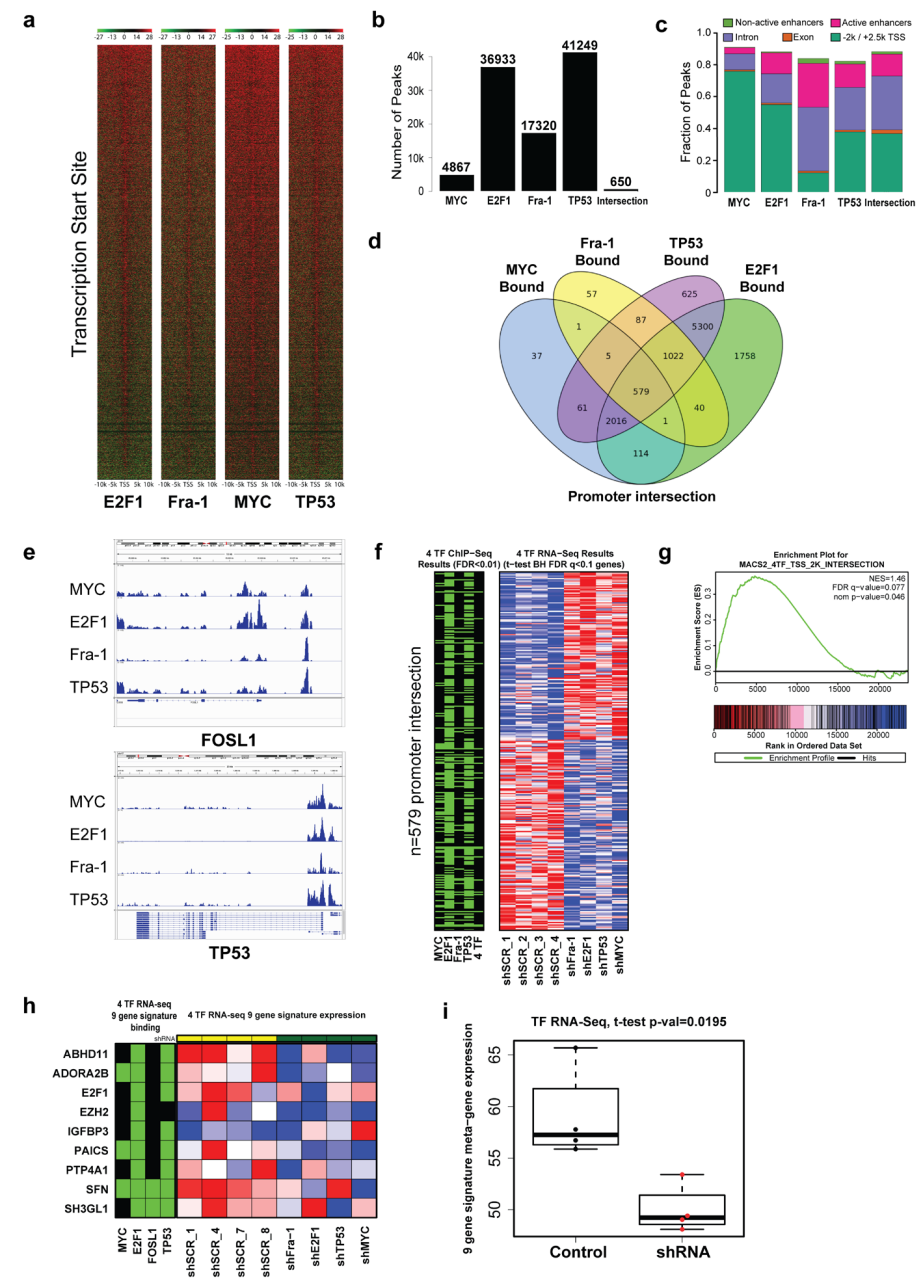
A substantial number of genes in the genome are bound, and regulated, by Fra-1, MYC, TP53 and E2F1 **a**. Coverage around the TSS with a +/−10kb window. Red signal corresponds with high levels of ChIP signal and green for input levels. Samples are ordered left to right: E2F1, Fra-1, MYC, and TP53 with the ranking of genes based on peak levels of E2F1. **b**. Histogram showing the number of peaks for the four transcription factors and the number of intersecting peaks for the four transcription factors. **c**. The association of peaks from the four TF with genetic regions: the -2k/+2.5k promoter region around the TSS, gene exons, gene introns, active enhancers, and non-active enhancers. **d**. A Venn diagram showing the overlap of genes with ChIP-Seq peaks in the promoter regions. **e**. IGV tracks for the four TFs for FOSL1 and TP53 (two examples of known interactions). **f**. A heat-map image of the differentially regulated genes in RNA-Seq data of control *vs*. knockdown for Fra-1, E2F1, TP53, and MYC along with a parallel heat-map (green/black) showing which of those genes have ChIP-Seq peaks in their promoters for the four TFs and the four-way overlap. Genes were selected for the heat-map by performing a *t*-test that compared the 8 control to the 8 knockdown samples (2 for each gene) and selecting the genes with a BH FDR < 0.1. The gene expression heat-map shows row normalized data for the FDR < 0.1 genes in the four control and four knockdown samples from batch 1 with red for high expression and blue for low expression. The ChIP-Seq heat-map shows genes with peaks in their promoters in green. **g**. GSEA enrichment plot in the RNA-Seq data for the 8 control and the 8 knockdown samples (2 for each gene) for the four TF using a gene-set with the 579 genes that had peaks within the promoter from all four TFs (Fra-1, E2F1, TP53, and MYC) (FDR < 0.01). **h**. A heat-map image of the nine-gene signature in RNA-Seq data of control *vs*. knockdown for Fra-1, E2F1, TP53, and MYC along with a parallel heat-map (green/black) showing results from ChIP-Seq for the four TFs that indicate which of the nine genes have ChIP-Seq peaks with FDR q < 0.01 in their promoter. **i**. Boxplot showing nine-gene-derived meta-gene expression in control and shRNA knockdown samples where meta-gene expression combines RNA-Seq values from the nine genes in the signature (p-value from *t*-test).

To test this hypothesis in an unbiased fashion, we determined by chromatin immunoprecipitation and sequencing (ChIP-Seq) where these four TFs reside across the genome. TFs such as these generally show a sharp enrichment near transcriptional start sites (TSS), which we confirmed in our dataset (Figure 5a). We then used the MACS2 algorithm to identify both statistically significant peaks for each TF (FDR < 0.01), and an overlapping set of 650 peaks common among these TFs, which was far greater than chance based on permutation testing (*p* < 1e-4) [18] (Figure 5b). While MYC, E2F1, and TP53 were most likely bound to gene promoters, Fra-1 was more frequently bound to introns or enhancers (Figure 5c). We noted simultaneous binding of all four TFs to promoters associated with 579 genes (Figure 5d, 5e, Supplementary Figure 6a). Similarly, we found coordinated binding of all four TFs to enhancers (441 genes) and active enhancers (412 genes) (defined by regions with both H3K4me1 and/or H3K27Ac using ChIP-Seq data on the parental MDA-MB-231 cell line [19]; Supplementary Figure 6b). These observations support the notion that Fra-1, MYC, E2F1, and TP53 display coordinated binding across the genome, and that these factors may work cooperatively at multiple levels to regulate gene expression.

To corroborate this concept, we performed RNA sequencing before and after depletion of each transcription factor. We found 1151 genes whose expression was significantly altered after knockdown by comparing knockdown samples for each of the four TFs to samples from control cells (600 down-regulated in knockdown and 551 up-regulated in knockdown; *t*-test FDR < 0.05; Figure 5f; Supplementary Figure 7). Using the 579-gene set of promoters bound by all four TFs, we found a statistically significant enrichment of this gene set in the expression signature associated with functional knockdown of each TF [20, 21] (FDR = 0.11; Figure 5g). These findings raised the possibility that each of these TFs might be functionally necessary for optimal expression of co-regulated target genes. In order to search support for this inference, we examined the effect of hairpin-mediated knockdown on each of the nine genes in our signature and found a coordinate decrease in expression (Figure 5h, 5i). We also noted that these nine genes had coordinated binding of these four TFs in the promoter regions (Figure 5h).

**Figure 6:**
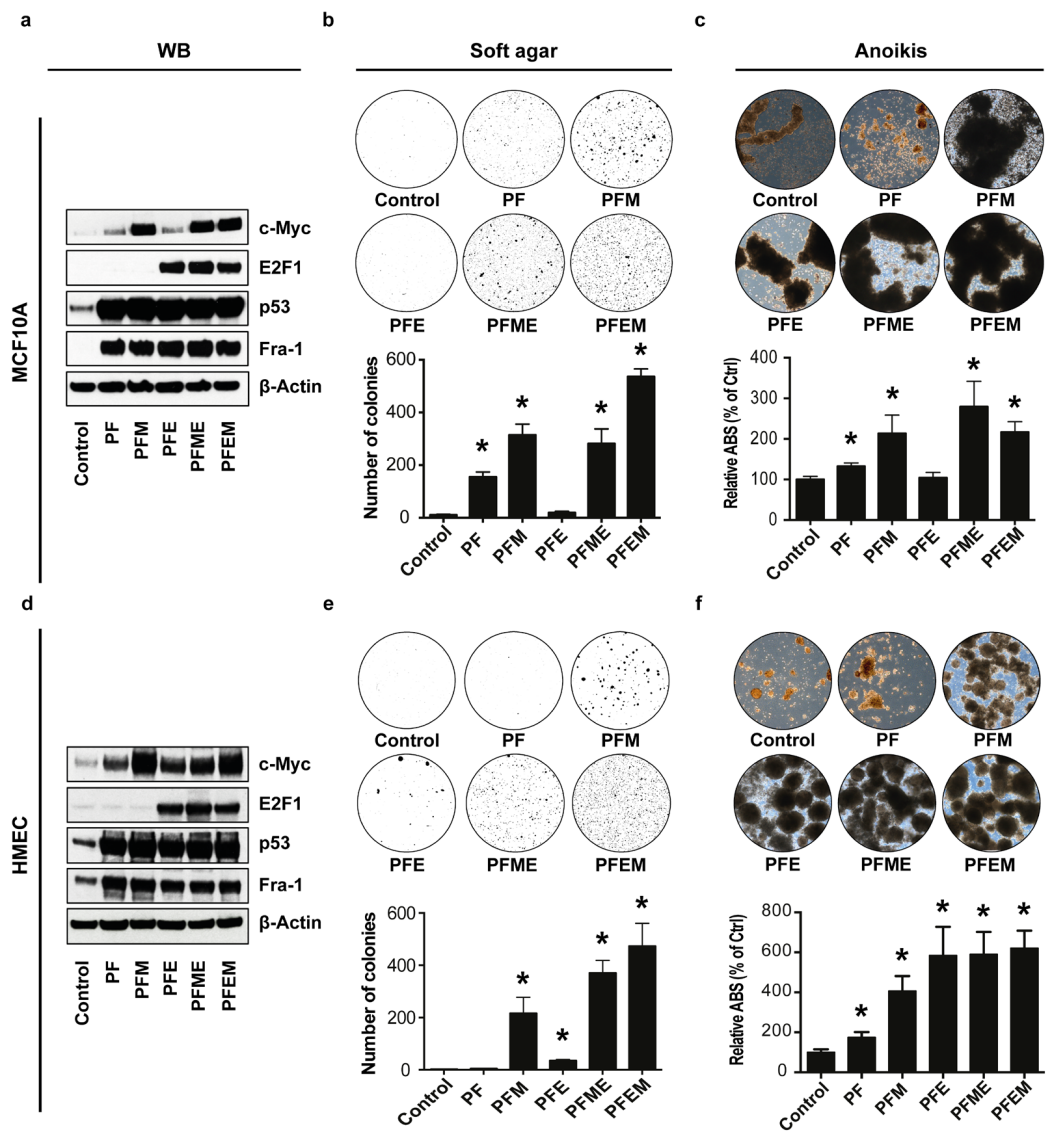
Fra-1, TP53, MYC and E2F1 cooperatively induce oncogenic transformation of non-malignant breast cancer and mammary epithelial cells.a./d Western blot analysis of protein expression in MCF10A and HMEC, respectively, expressing a control vector, mutp53 and Fra-1 (PF), mutp53, Fra-1 and c-Myc (PFM), mutp53, Fra-1 and E2F1 (PFE), mutp53, Fra-1, c-Myc and E2F1, or mutp53, Fra-1, E2F1 and c-Myc. β-Actin was used as a loading control. **b**./**e**. Representative images of colony formation in soft agar by MCF10A and HMEC cells, respectively, expressing a control vector or the combinations of transcription factors as described above. Below the images, bar graphs show the quantification of the corresponding assays. **c**./**f**. Representative images of anoikis-resistant colonies of MCF10A and HMEC cells, respectively, expressing control vector or combinations of transcription factors as described in a./d., along with quantifications. (*n* = at least 2 independent experiments, containing at least 2 technical replicates each, error bars: SEM, *p < 0.05 compared to control, following a *t*-test).

These data showed that the nine-gene set is required for breast cancer growth and aggressiveness, and that this correlates with binding of a substantial number of genes in the genome by the four transcription factors associated with the network, namely, Fra-1, MYC, mutTP53 and E2F1. This further raised the possibility, conversely, that these transcription factors might be sufficient to endow non-oncogenic mammary epithelial cells with an oncogenic phenotype. To test this hypothesis, we introduced into MCF10A breast epithelial cells and primary human mammary epithelial cells (HMEC) cassettes driving the expression of each of these transcription factors (Figure 6a, 6d). Indeed, ectopic co-expression of Fra-1, MYC, mutTP53 and E2F1 was sufficient to stimulate massive growth of these cells in soft agar (Figure 6b, 6e). Another *in vitro* hallmark of oncogenic transformation is suppression of anoikis [22] [23]. Consistently, this gene set also strongly stimulated survival under detachment conditions (Figure 6c, 6f). Downscaling experiments showed that co-expression of three, and sometimes even two, transcription factors was already sufficient to mediate soft agar growth and anoikis resistance, albeit to a lesser extent than was seen for the four factors. Thus, coordinate expression of the four transcription factors associated with the poor prognosis nine-gene network is sufficient to bring about oncogenic changes in non-oncogenic breast epithelial cells, at least *in vitro*.

## DISCUSSION

To our knowledge, this study is the first systematic analysis to functionally annotate the contribution of individual genes contained in a poor prognostic genetic signature. Whereas prognostic classifiers have proven useful in identifying good-prognosis patients who should be spared from adjuvant chemotherapy [1, 2, 4, 24–26], they have not yet been explored in guiding the best therapeutic options for poor-prognosis patients. The recent prospective validation of two breast cancer signatures [7, 8] not only emphasizes their clinical utility but also highlights their potential biological value, in that they may harbor prognostic genes that, in fact, contribute to the aggressive nature of the disease. Therefore, it is important to functionally annotate, in a systematic fashion, poor prognosis signature genes. Our results suggest that the Fra-1 genetic classifier may be used for designing personalized therapies. The nine genes, each of which we demonstrate to have a critical contribution to breast cancer outgrowth and aggressiveness, encode several proteins that are amenable to targeted intervention. For example, as we and others have shown previously, pharmacological inhibition of the adenosine receptor ADORA2B strongly inhibits breast cancer in mice [9, 27, 28], as do monoclonal antibodies directed against PTP4A1 (PRL1) [29]. An inhibitor of EZH2 expression (DZNeP) has anti-tumor and anti-invasive activities against breast and other cancers [30]. Also the activity of enzymes such as ABHD11 and PAICS ought to be inhibited by small molecules, which we are currently exploring. Furthermore, full understanding of the molecular mechanism by which these factors contribute to breast cancer may yield additional therapeutic opportunities. Our data raise the intriguing possibility to develop companion diagnostics, that is, to use the Fra-1 classifier to identify those patients who are associated with a poor prognosis, and treat them with one or more inhibitory agents targeting the nine-gene set. As this signature integrates prognostic power with therapeutic targets, it may contribute towards a more personalized management of poor-prognosis breast cancer.

## MATERIALS AND METHODS

### Gene silencing in LM2 cells

LM2 cells (subline#4173 [12], a kind gift of J. Massagué, New York) were cultured in DMEM (Life Technologies) supplemented with 10% FCS (Greiner bio-one), 2 mM glutamine, 100 units ml^−1^ penicillin, and 0.1 mg ml^−1^ streptomycin (Gibco). Gene silencing in LM2 cells was performed using pLKO.1 vectors from the TRC library (Sigma). Around 5 shRNAs for each gene were tested and the 2 most efficient ones were selected for further studies. As a negative control, vector without insert was used. Alternatively, vectors containing a scrambled sequence or an shRNA targeting luficerase gene (Sigma) were used. References of pLKO.1 vectors used in experiments are listed in Supplementary Table 1. Lentiviral particles were produced by transfection of the HEK293T cell line and supernatants were collected 48 h after transfection. Supernatants were used to infect sub-confluent cultures in the presence of 5 μg ml^−1^ polybrene overnight. Puromycin (2 μg ml^−1^) was then used to select for stable cell lines.

### RNA isolation and qRT-PCR

RNA was extracted from exponentially growing cells using TRIzol reagent (Life Technologies). Total RNA was DNase-treated with RQ1 RNase-Free DNase (Promega). Reverse transcription was performed using Superscript II first strand kit (Invitrogen). qRT-PCR was performed with the SYBR Green PCR Master Mix on a StepOne Real-Time PCR System (Applied Biosystems). Primer sets used are listed in Supplementary Table 2. mRNA levels were normalized using β-Actin mRNA levels.

### *In vivo* experiments

All animal experiments were done in accordance with a protocol approved by the NKI Institutional Animal Experiment Ethics Committee. Female Balb/c nude mice aged 6-8 weeks were used for all xenografting experiments. For experimental lung metastasis assays, 1 × 10^5^ or 2 × 10^5^ viable cells were resuspended in 150 μl of PBS and injected into the lateral tail vein. When GFP-labeled LM2 cells were used, mice were sacrificed 5 weeks after cells inoculation by CO_2_ asphyxiation. Lungs were subsequently dissected and imaged within 2 hours by fluorescence microscopy. Images were taken with the same intensities and exposure times, and the mean fluorescence intensity per surface area was quantified using ImageJ software (http://rsb.info.nih.gov/ij/download.html). When luciferase-labeled LM2 cells were used, mice were injected intraperitonally with D-Luciferin (Caliper Life Sciences), 150 μg/g body weight, and anesthetized with isoflurane. Images were acquired 15 min after D-Luciferin injection with a cryogenically cooled IVIS system using LivingImage acquisition and analysis software (Xenogen Corp.). Photon flux was determined by using a rectangular region encompassing the thorax of the mouse. These values were normalized to the values obtained immediately after xenografting of the cells for each mouse. Mice were sacrificed when clinical symptoms became apparent. Orthotopic tumor growth was measured by injecting 1 × 10^6^ viable cells in 50 μl of a 1:1 mixture of PBS and growth-factor-reduced Matrigel (BD Biosciences) into the 4^th^ mammary fat pad in each flank. Primary tumor growth rates were analyzed by measuring at regular time points the tumor length (*L*) and width (*W*), and tumor volume (*V*) was estimated using the formula *V* = *LW*^2^/2. Mice were sacrificed when the tumor length reached a size of ≥15 mm or when the tumors started to ulcerate.

### *In vitro* proliferation, soft agar and anoikis assays

For proliferation assays LM2 cells (2× 10^4^ cells) were seeded in 6 well plates at day 0. At regular time points cells were trypsinized and the number of cells in each well was calculated using a CASY cell counter (Innovatis). Soft agar assays were performed as described in [9]. Colonies were imaged using a GelCount Colony Counter (Oxford Optronix) and images were quantified using ImageJ software. Anoikis assays were performed by seeding 5×10^4^ cells into ultra-low cluster 6 well plates where they were left to grow out for 3 weeks. Afterwards, images were taken and total protein content per well was quantified with a Bradford protein assay (Bio-Rad).

### Microarray and gene-expression signature analysis

Full description of the methods and results for each experiment is available athttp://www.ebi.ac.uk/microarray-as/aer/#ae-main [0] (accession numbers E-MTAB-1230 and E-NCMF-27 for MDA-MB-231 and LM2 data, respectively). The Agilent probes that were significantly up- or down-regulated by both Fra-1 shRNAs (*p* < 1.10^−6^) were selected and mapped to the corresponding Affymetrix U133A probes using Martview from BioMart (http://www.biomart.org/index.html). We selected a single Affymetrix HGU-133A probe for each Entrez ID based on the Affymetrix algorithm probe extension, favoring ‘_at’ over ‘_x_at’ over ‘_s_at’. Expression of remaining duplicate probes were averaged, resulting in a 1140 and 1234 probe set for MDA-MB-231 and LM2 data, respectively.

For generation of the gene-expression signatures, we collected six publicly available datasets based on Human Genome HGU-133A Affymetrix arrays from NCBI's Gene Expression Omnibus (GEO,http://www.ncbi.nlm.nih.gov/geo/) with the following identifiers: GSE6532 [31], GSE3494 [32], GSE1456 [33], GSE7390 [34] and GSE5327 [35]. The Chin et al. data set was downloaded from ArrayExpress (http://www.ebi.ac.uk/, identifier E-TABM-158).

To ensure comparability between the different datasets, they were all subjected to the same pre-processing procedure. Microarray quality-control assessment was carried out using the R AffyPLM package (Bioconductor,http://www.bioconductor.org). We applied the Relative Log Expression (RLE) and Normalized Unscaled Standard Errors (NUSE) tests. Chip pseudo-images were produced to assess artefacts, and 1 to 5% of the arrays of the datasets did not pass the quality control tests. Selected arrays were normalized according to a 3-step procedure using the RMA expression measure algorithm (http://www.bioconductor.org): RMA background correction convolution, median centering of each gene across arrays separately for each data set and quantile normalization of all arrays. Out of the 947 unique collected microarray samples of sufficient quality, 509 had Distant Metastasis Free Survival (DMFS) data available. We employed these samples as training set. From the experimental Fra-1 signature of 1140 and 1234 unique probes, those probes were extracted that exhibited a P-value *P* < 0.1 (log-rank test) on the training set. This resulted in a subset of 445 and 447 probes for MDA-MB-231 and LM2, respectively.

### Clinical outcome and tumor grade analysis

For Figure 1b, Figure 2c, 2d, and Supplementary Figure 4, the datasets used and their breakdown by molecular sub-type (when available) are as given in Figure 3b and 3c of Desmet *et al*. [9]. The Kaplan-Meier curve, Cox hazard-ratio and p-value were computed as for Supplementary Figure 10 of Desmet *et al*. [9]. We used the following method to generate Supplementary Figure 4. From all publically available breast cancer tumor expression databases known to us at the time of analysis we selected those datasets that had at least 10 annotated grade III and 10 annotated grade I tumors. We ensured that only one tumor was used from each patient. This yielded the datasets given in Supplementary Table 3 and Supplementary Figure 4. For each dataset the standardized mean difference was estimated by the following estimate of the unbiased estimator

$$g^{*}\approx \left(1-\frac{3}{4\left(n_{1}+n_{2}\right)-9}\right)g$$

where

$$g=\frac{{\bar{x}}_{1}-{\bar{x}}_{2}}{s^{*}}$$

and

$$s^{*}=\sqrt{\frac{(n_{1}-1)s_{1}^{2}+(n_{2}-1)s_{2}^{2}}{n_{1}+n_{2}-2}}$$

The standard error was then estimated by

$$\sigma ˆ(g^{*})=\sqrt{\frac{n_{1}+n_{2}}{n_{1}n_{2}}+\frac{{\left(g^{*}\right)}^{2}}{2(n_{1}+n_{2}-3.94)}.}$$

The standardized mean difference and standard error for each of the ten datasets were then combined using a random effects model. A p-value and 95% confidence interval was computed from the combined standardized mean difference and standard error using the assumption that the estimate of the standardized mean difference has a normal distribution [36].

### Chromatin immunoprecipitation

Cells were harvested by crosslinking with 1% formaldehyde in cell culture medium for 15 min at room temperature. After quenching with the addition of 125 mM glycine for 5 min at room temperature, the cells were washed twice with ice cold PBS. After aspiration of all liquid, pellets consisting of ∼10^7^ cells were flash frozen and stored at −80 °C. Fixed cells were thawed and sonicated to obtain chromatin fragments of ∼200 to 700 bp with a Branson 250 Sonifier. Solubilized chromatin was immunoprecipitated with ∼5 μg antibody against c-Myc (Santa Cruz; sc-764), Fra-1 (Santa Cruz; sc-183), E2F1 (Millipore; 05-379) and p53 (BD-Pharmingen; 554294). Immunoprecipitation was performed retaining a fraction of input ‘whole-cell extract’ as a control. Antibody-chromatin complexes were pulled-down using Dynabeads Protein G, washed and then eluted. After crosslink reversal and proteinase K treatment, immunoprecipitated DNA was extracted with phenol, precipitated in ethanol and treated with RNase. ChIP DNA was quantified by fluorometry using the Qubit assay (Invitrogen). The Western blot results shown in Figure 6 and Supplementary Figure 7 were achieved using the same antibodies as described here.

### Library preparation and illumina sequencing

For each ChIP or control sample, ∼5 ng of DNA was used to generate a standard Illumina sequencing library. Briefly, DNA fragments were end-repaired using the End-It DNA End-Repair Kit (Epicentre), extended with a 3′ ‘A’ base using Klenow (3′ 5′ exo-, 0.3 U μl^−1^, NEB), ligated to standard Illumina adapters (75 bp with a ‘T’ overhang) using DNA ligase (0.05 U μl^−1^, NEB), gel-purified on 2% agarose, retaining products between 275 and 700 bp, and subjected to 18 PCR cycles. These libraries were quantified by fluorometry and evaluated by quantitative PCR to confirm representation and specific enrichment of DNA species. Libraries were sequenced in one or two lanes on the HiSeq 2000 using standard procedures for cluster amplification and sequencing by synthesis.

### ChIP-Seq data analysis

Sequencing read quality was examined using FastQC (http://www.bioinformatics.babraham.ac.uk) at three stages in the analysis pipeline: on the raw data, after trimming, and after duplicate reads were removed. Trimming of low quality reads and clipping of sequencing adapters was done using the program Trimmomatic [37] and all reads shorter than 35bp after trimmer were dropped. Reads were aligned to a masked genome (hg19) using Bowtie, only keeping uniquely mapping reads, with no mismatches in the first 45bp (M = 1, N = 0, L = 45) [38]. Bam to Sam file conversion was done with SamTools [39], and duplicate reads were removed using Picard-tools (http://picard.sourceforge.net). ChIP-Seq heatmap plots of Figure 5a were generated through the use of NGSPlot [40]. Peaks were called using MACS2 [18] with the False Discovery Rates (FDR) q < 0.01 with a distribution across the factors as shown in Figure 5b and Figure 5c shows the distribution across genomic regions. The MACS2 algorithm utilizes a dynamic Poisson distribution to capture local biases in the genomic sequence, which allows for a sensitive and robust prediction of peaks. The IGV browser [41] was used to visually check called peaks and produce the ChIP-Seq traces in Figure 5e and Supplementary Figure 6a. Peaks were assigned to genes using PeakAnnotator in the PeakAnalyzer package where positive genes were determined by the presence of a peak in a -2kb and +2.5Kb window around the transcription start site (TSS) [42]. The Venn diagrams of Figure 5d and Supplementary Figure 6b were made with the R package VennDiagram.

### RNA sequencing

In parallel with the ChIP sample preparation, ∼5 × 10^6^ cells were harvested and RNA isolation was done in TRIzol reagent. Libraries were prepared for sequencing using standard Illumina TrueSeq protocols. Libraries were pooled and sequenced 51 bp on a HiSeq2000. After sequencing, basecalling and demultiplexing have been performed using the standard casava pipeline.

### RNA-Seq data analysis

Gene expression values were derived from RNA-Seq data for MDA-MB-231 derivative LM2 cells treated with shRNA for Fra-1, E2F1, TP53, MYC, or scrambled (two biological replicates for each gene and controls). FastQC was used to evaluate read quality on raw RNA-Seq reads and trimmed reads. Trimming of low quality reads and clipping of sequencing adapters was done using the program Trimmomatic [37] and all reads shorter than 35bp after trimming were dropped. Reads were aligned to the hg19 reference genome with TopHat [43] version 2.0.8. Bam to Sam file conversion, sorting, indexing, and file merging was done with SamTools [39]. FPKM values (Fragments per Kilobase of transcript Per Million mapped reads) were calculated by Cufflinks [43] version 2.1.1. FPKM data was loaded into a matrix in R and a variation filter was applied to remove genes with less than 1.5 fold minimum variation and 1 minimum absolute variation (leaving 12239 out of 23615 genes). A *t*-test was then performed to find genes significantly varying between scrambled and TF knockdown samples and corrected for multiple hypothesis testing using the Benjamini-Hochberg step-up FDR-controlling procedure [44]. Genes with a Benjamini-Hochberg FDR value < 0.1 were selected leaving 295 genes (157 down in knockdown and 138 up in knockdown). The red-blue heat map in Figure 5i was produced after scaling each row of data to a zero to one range. A parallel heat map with the same genes was produced for ChIP-Seq data using green to indicate genes that had peaks for each ChIP-Seq factor with FDR < 0.01 within a window of -2kb / +2.5kb of the gene's TSS. The red-blue heat-map of Figure 5h was produced for the genes of the nine-gene signature after scaling each row of data to a zero to one range. A parallel heat map with the same nine genes from the nine-gene signature was produced with the ChIP-Seq data using green to indicate genes that had peaks for each ChIP-Seq factor with FDR < 0.01 within a window of -2kb / +2.5kb of the gene's TSS. The box plot of Figure 5i was produced by calculating meta-gene values for the control and shRNA knockdown samples where the meta-gene value for the nine-gene signature is found by taking the mean of the RNA-Seq values for all nine genes in the signature in each sample.

In order to test the correlation of the nine-gene signature with proliferation, TCGA RNASeqV2 data for breast cancer (BRCA) was downloaded from TCGA data matrix access portal (http://cancergenome.nih.gov/). Proliferation score came from the TCGA BRCA paper by Ciriello et al. [45], comprising data for 817 of the 1093 TCGA BRCA RNASeqV2 samples. The meta-gene value for the nine-gene signature is found by taking the mean of the log2 RNASeqV2 values for all nine genes in the signature in each sample. Supplementary Figures 3b for all subtypes of breast cancer samples (817) and 3c for the TNBC subset (116 samples) show Pearson correlation between the meta-gene and Ciriello et al. [45]. Proliferation Score from a correlation test (cor.test() in R).

### Gene set enrichment analysis

Gene Set Enrichment Analysis (GSEA) [20,21] was used to evaluate the association of genes bound by the 4 transcription factors with regulation and produce the enrichment plot of Figure 5g. A gene set was made out of the 579 genes that had peaks with FDR < 0.01 associated with the TSS for all four TFs (Fra-1, E2F1, TP53, and MYC) and tested for enrichment in the RNA-Seq data of control *vs*. knockdown for Fra-1, E2F1, TP53, and MYC (8 *vs*. 8 samples). GSEA was run with 1000 permutations of the phenotype using signal-to-noise to rank genes.

### Overexpression of transcription factors in MCF10A and HMEC

Overexpression constructs were made by performing PCR on LM2 (whole) cDNA to amplify *TP53*, *FOSL1*, *MYC* and *E2F1*. Primers were so designed that each of the amplicons was flanked by a NotI site on the 5′-forward end and a BamHI site on the 5′-reverse end. The cDNA was then cloned into a set of lentiviral vectors, each with a different selection marker. These vectors were: pHAGE2-EF1aFull-rtTA-IRES-Puro-W (where the rtTA is replaced with one of the cDNAs), pHAGE2-EF1a-ZsGreen-IRES-Blasticidin-W (ZsGreen is replaced), pHAGE2-FullEF1a-DsRedExpress-IRES-ZsGreen-W (DsRedExpress is replaced) and pHAGE2-FullEF1a-ZsGreen-IRES-dTomato-W (ZsGreen is replaced), which were a kind gift of Dr. Gustavo Mostoslavsky. Lentivirus production and infection of MCF10A/HMEC cells was done as described above. ZsGreen and/or dTomato expression was used to select populations by FACS (MoFlo Asterios, Beckman-Coulter). Cells expressing puromycin and/or blasticidin resistance cassettes were selected with 1 μg ml^−1^ and 5 μg ml^−1^, respectively.

## SUPPLEMENTARY MATERIALS FIGURES AND TABLES


