# Supplementary material for: Systematic functional perturbations uncover a prognostic genetic network driving human breast cancer
Source: Oncotarget. 2017 Mar 15;8(13):20572–87. doi: 10.18632/oncotarget.16244 (PMC5400527; doi:10.18632/oncotarget.16244)
Supplement: Supplementary file 1 [file oncotarget-08-20572-s001.pdf]

Systematic functional perturbations uncover a prognostic genetic network driving human breast cancer

Supplementary Material

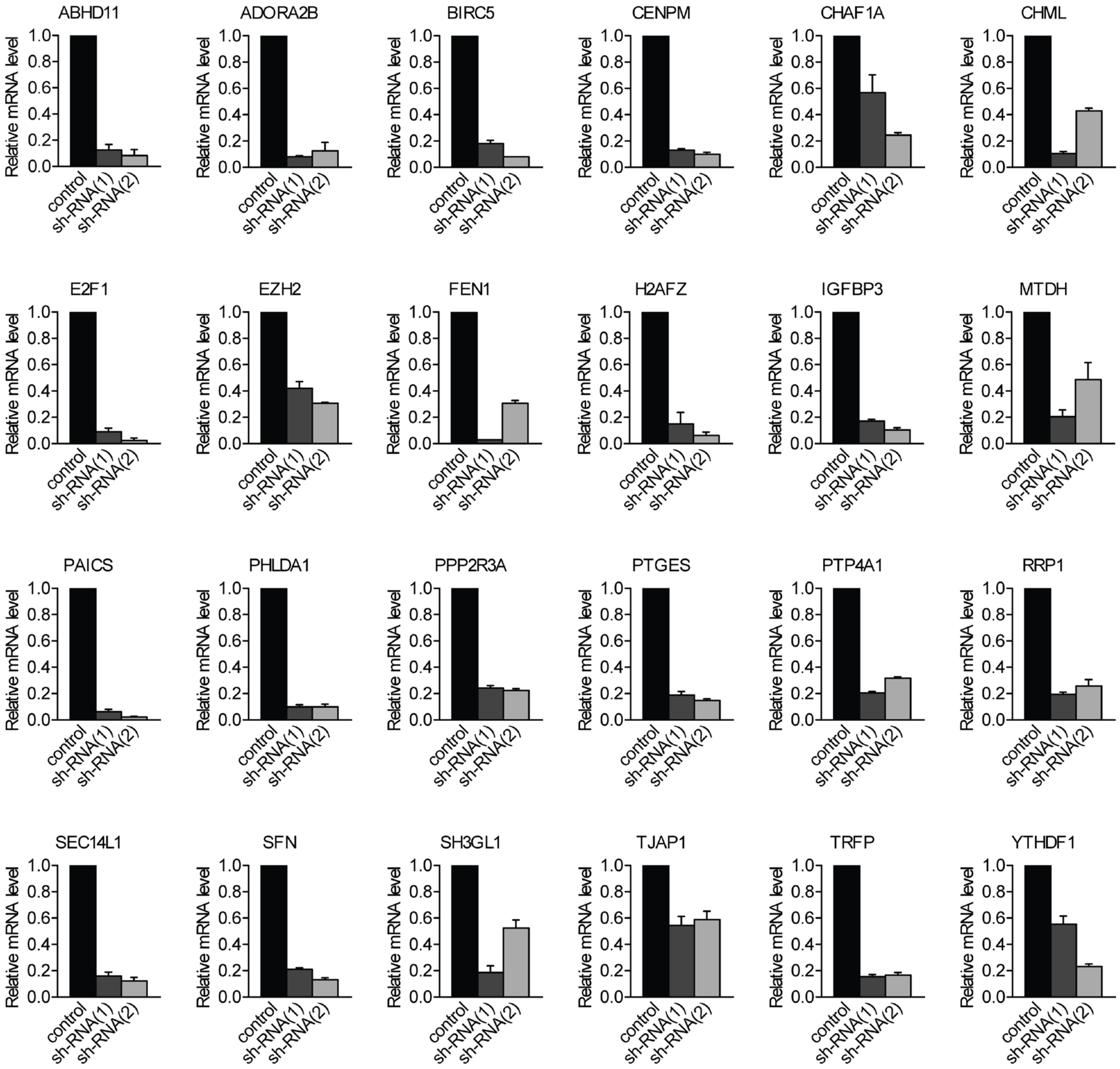

Supplementary Figure 1: Confirmation of knockdown of 24 poor prognosis genes. Expression levels of the indicated genes in LM2 cell expressing two independent shRNAs targeting the corresponding genes measured by quantitative RT-PCR (n=3, error bars: S.D.).

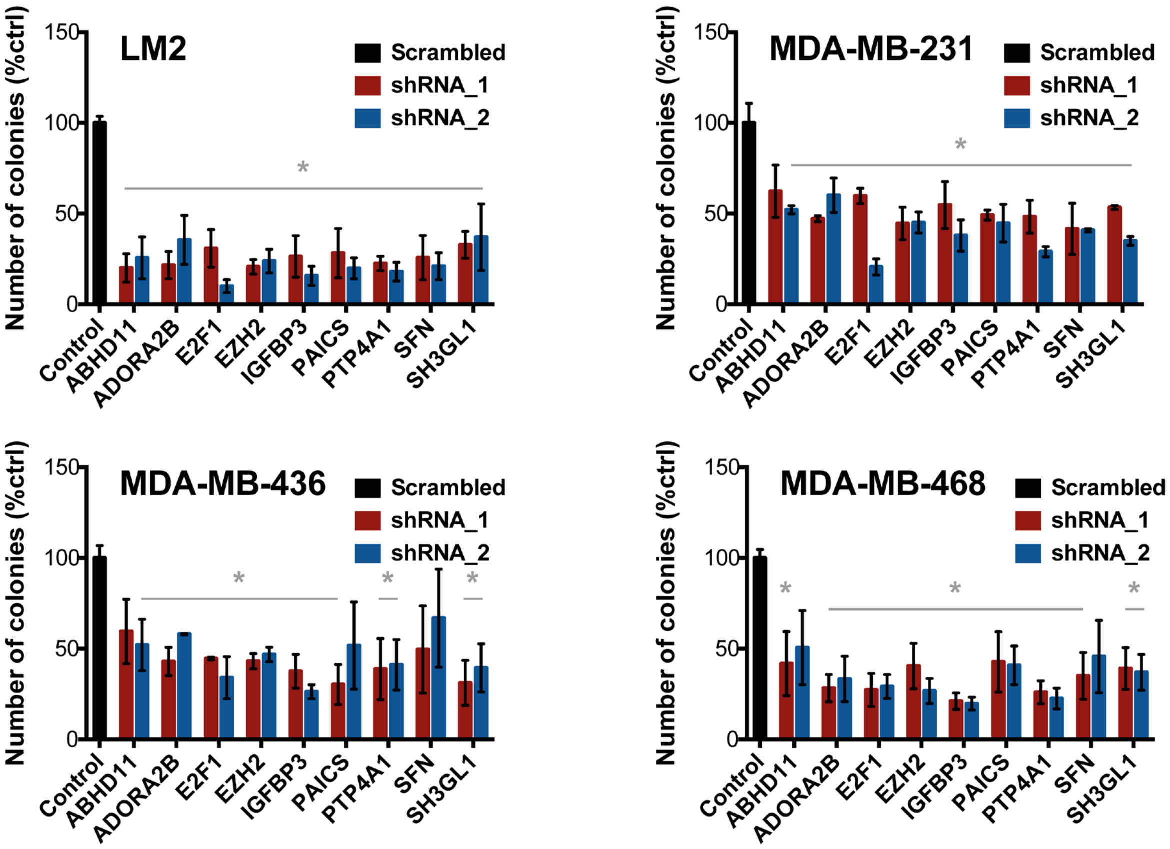

Supplementary Figure 2: The 9 genes play a critical role in the aggressive phenotype of multiple breast cancer cell lines. Normalized colony count of soft agar assays, performed in 4 different cell lines, each with individual knockdown of the 9 genes by 2 independent shRNAs (n=2 independent experiments, having each at least 2 technical replicates, error bars: SEM, *p<0.05 compared to control, following a t-test).

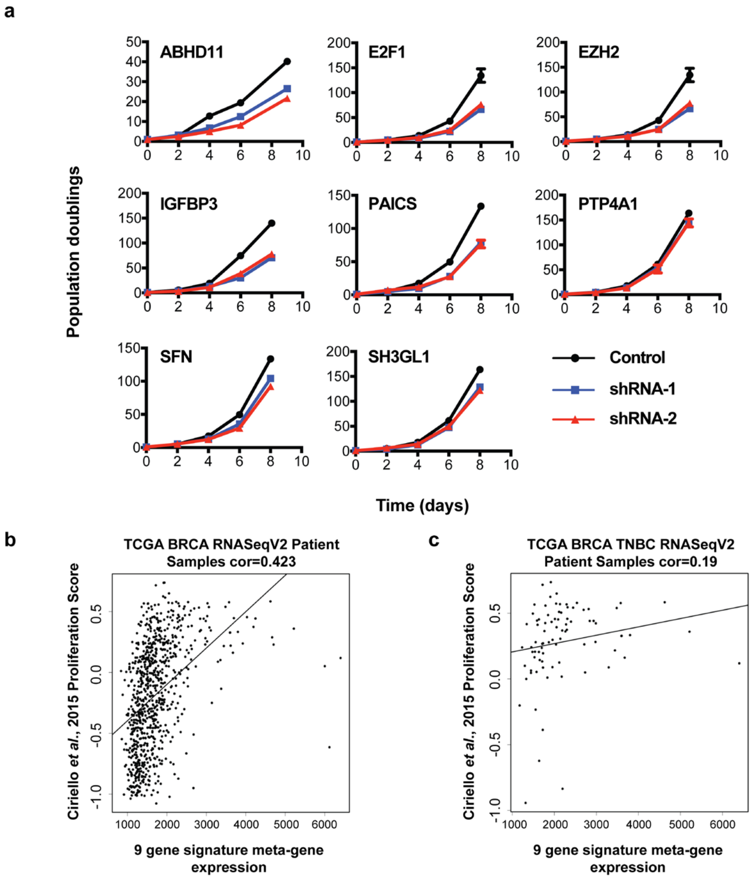

Supplementary Figure 3: Depletion of the components of the nine genes has little effect on cell proliferation *in vitro*. a. In vitro proliferation curves of LM2 cells expressing a control vector or two independent shRNAs directed against the indicated genes (n=3, error bars: SD). b. Scatter plot showing the association between a meta-gene for the nine-gene Fra-1 signature and the proliferation score in 817 TCGA BRCA RNASeqV2 samples including all subtypes of breast cancer. c. Scatter plot showing the association between a meta-gene for the nine-gene Fra-1 signature and the proliferation score in just the 116 TNBC samples in the TCGA BRCA RNASeqV2 data.

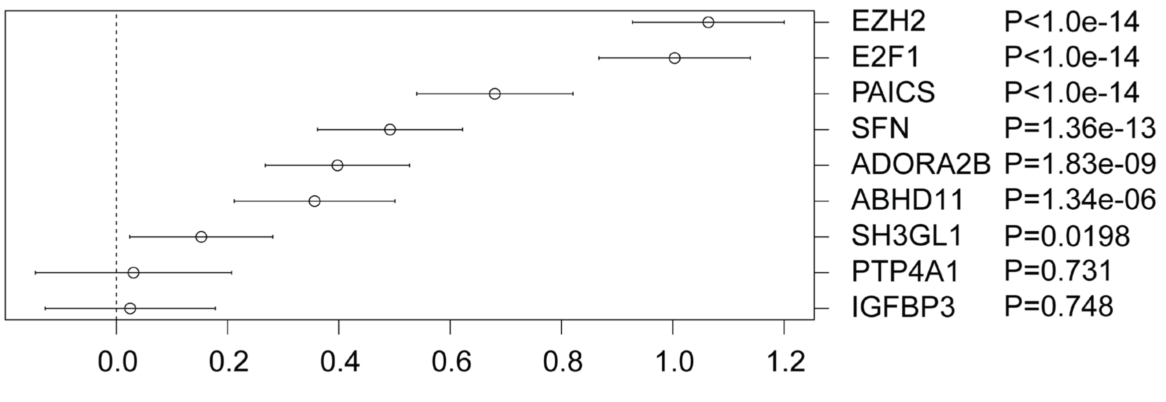

Supplementary Figure 4. Comparison of the expression of components of the nine-gene set in grade III versus grade I breast tumors. **X-axis values are estimates of the standardized mean difference, $(\mu_{III} - \mu_{I})/\sigma$, where μ_{III} is the mean expression of the gene in grade III tumors, μ_{I} is the mean expression of the gene in grade I tumors, and σ is the standard deviation of the gene's expression in grade III and grade I tumors.** Estimates are derived from a meta-analysis of ten breast cancer datasets representing 1076 unique patients. Error bars are 95% confidence intervals. P-values are for the null hypothesis $\mu_{III} = \mu_{I}$.

a

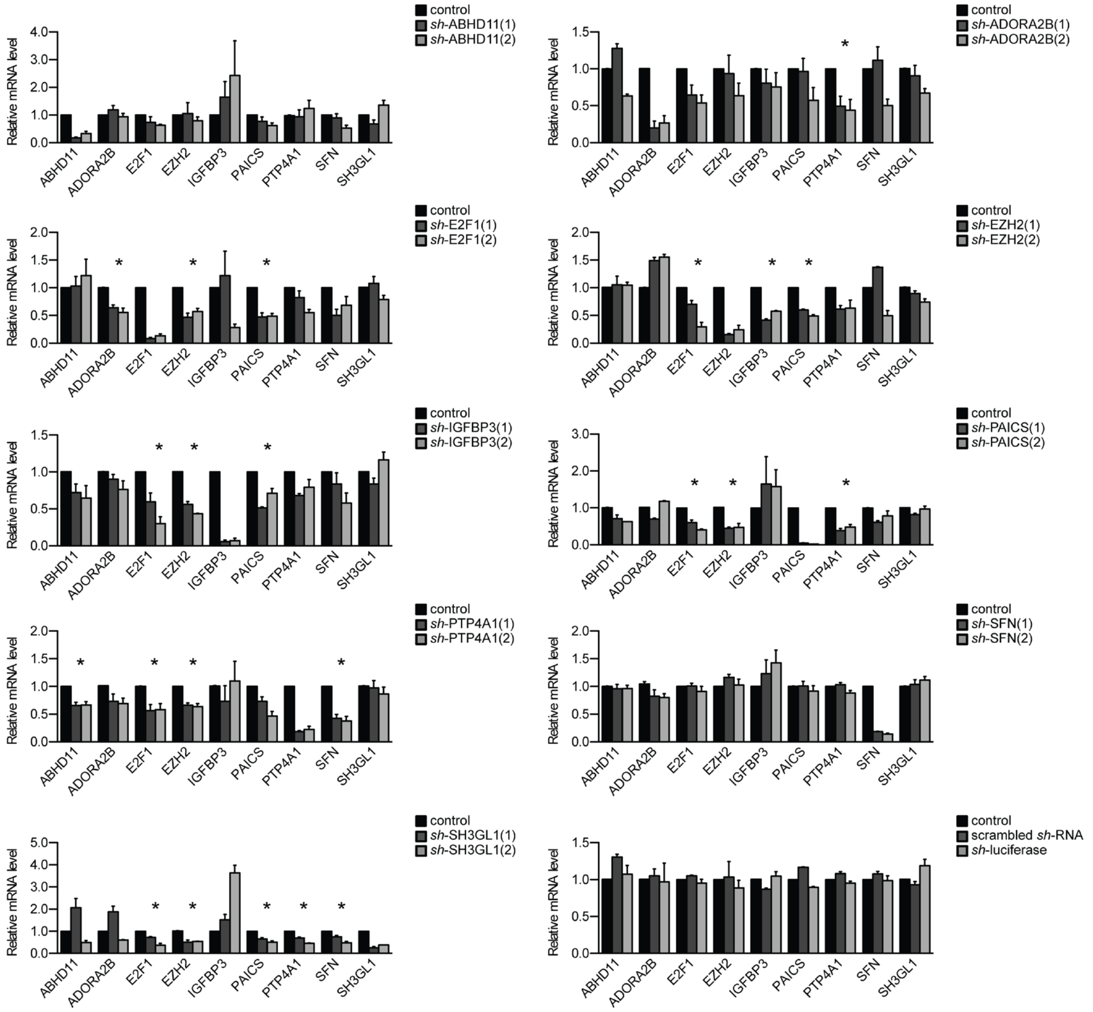

b

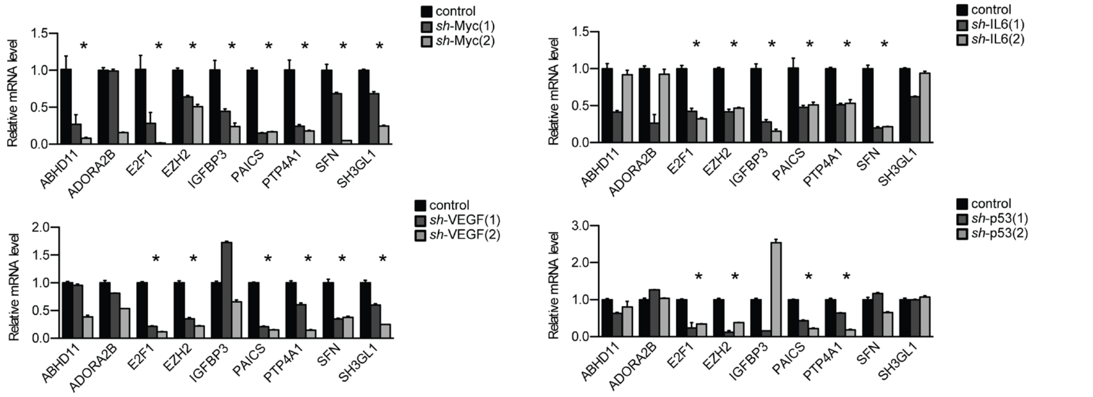

Supplementary Figure 5: Depletion of single genes from the nine-gene set affects expression levels of the other genes. a. Relative expression levels of components of the nine-gene set in LM2 cell expressing a control vector or two independent shRNAs targeting the indicated genes, as measured by quantitative RT-PCR (results are average of 3 independent experiments, error bars: S.E. *p<0.05, following a one-way ANOVA test). b. As for a, for depletion of MYC, VEGF, IL6 and p53.

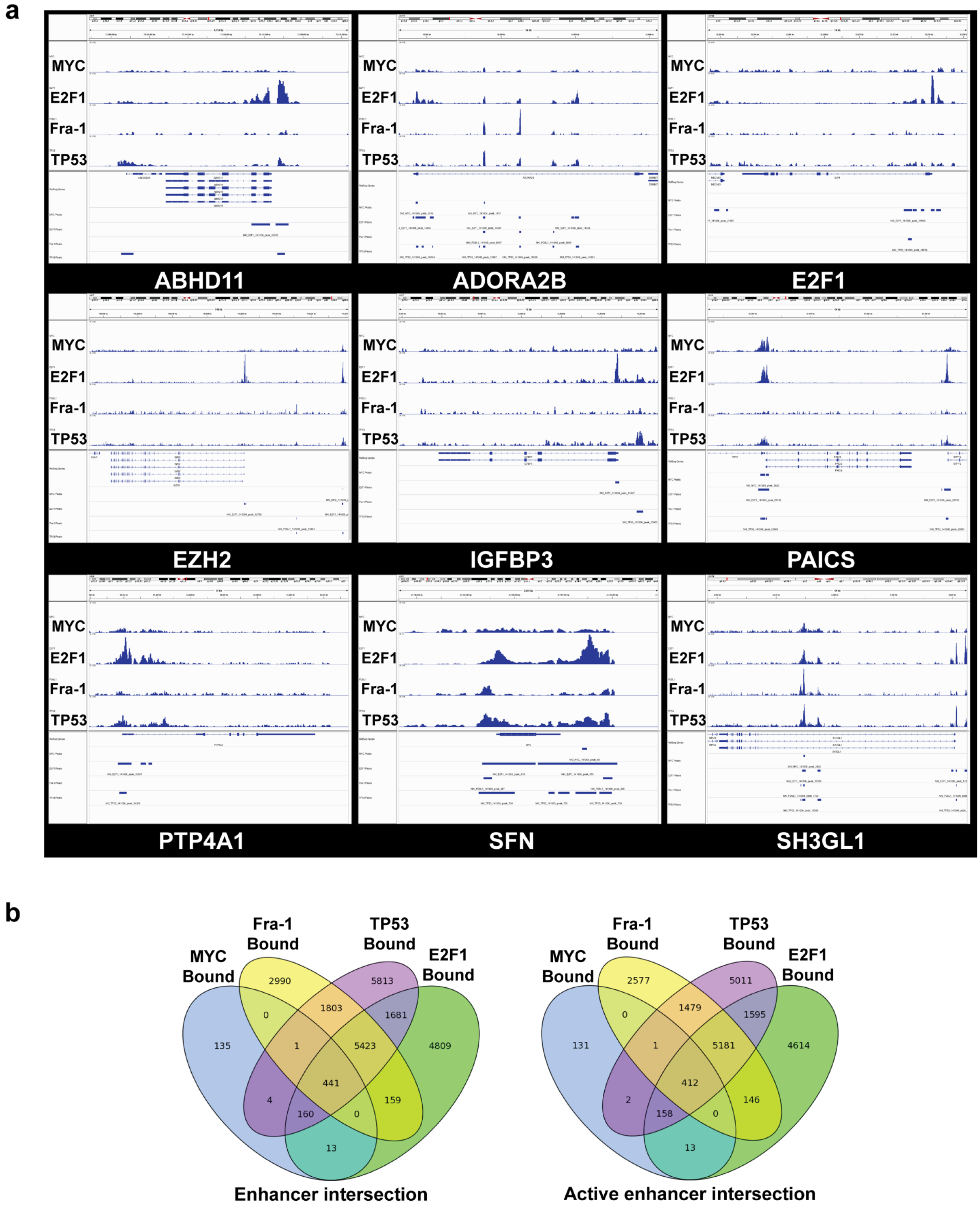

Supplementary Figure 6: The four transcription factors show overlapping binding peaks on the promoters of the nine signature genes and bind coordinately to (active) enhancers genome-wide. a. IGV tracks showing ChIP-Seq binding peaks for the four transcription factors to the nine signature genes. b. Two Venn diagrams showing the overlap in ChIP-Seq peaks of enhancer and active enhancer regions, respectively.

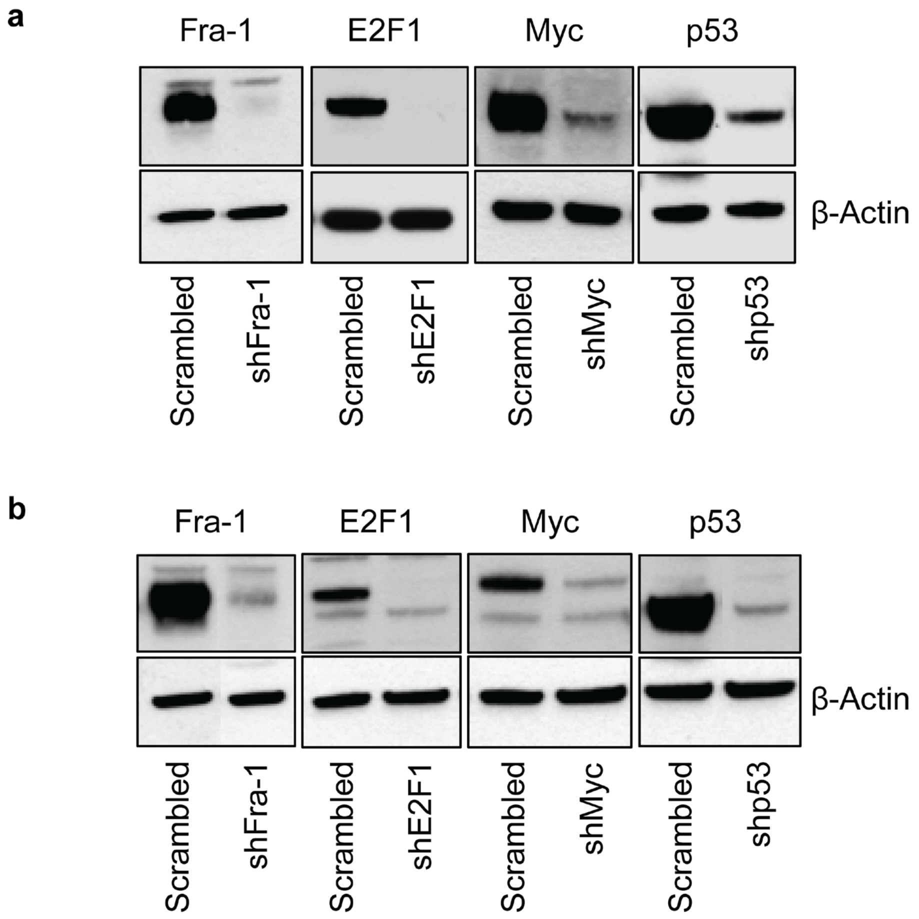

Supplementary Figure 7: Confirmation of knockdown of the four Transcription Factors. Western blot analysis in protein lysates of LM2 cells expressing a control vector or shRNA targeting indicated TF, used for a. ChIP-seq and simultaneously for the first of 2 biological replicates for RNA-seq and b. The second biological replicate for RNA-seq.

Supplementary Table 1. TRC library sh-RNA index

Gene	sh-RNA(1)	sh-RNA(2)
ABHD11	TRCN0000050728	TRCN0000050732
ADORA2B	TRCN0000065335	TRCN0000065336
AURKB	TRCN0000000776	TRCN0000000777
BIRC5	TRCN0000073719	TRCN0000073720
CENPM	TRCN0000150803	TRCN0000156094
CHAF1A	TRCN0000074275	TRCN0000074277
CHML	TRCN0000065188	TRCN0000065191
E2F1	TRCN0000000250	TRCN0000010328
EZH2	TRCN0000040074	TRCN0000040076
FEN1	TRCN0000049729	TRCN0000049732
FOXM1	TRCN0000015544	TRCN0000015546
H2AFZ	TRCN0000072583	TRCN0000072585
IGFBP3	TRCN0000072511	TRCN0000072512
IL6	TRCN0000059203	TRCN0000059207
MCM10	TRCN0000245425	TRCN0000245428
MCM2	TRCN0000019509	TRCN0000019513
MTDH	TRCN0000151467	TRCN0000152120
MYC	TRCN0000039641	TRCN0000010391
PAICS	TRCN0000045777	TRCN0000045774
PHLDA1	TRCN0000150307	TRCN0000155644
PPP2R3A	TRCN0000006872	TRCN0000006874
PTGES	TRCN0000045933	TRCN0000045936
PTP4A1	TRCN0000002919	TRCN0000002922
RRP1	TRCN0000129053	TRCN0000128839
SCD	TRCN0000056614	TRCN0000056616
SEC14L1	TRCN0000060149	TRCN0000060150
SFN	TRCN0000040130	TRCN0000010287
SH3GL1	TRCN0000083924	TRCN0000083925
SMTN	TRCN0000123230	TRCN0000123233
TJAP1	TRCN0000150562	TRCN0000156011
TP53	TRCN0000003754	TRCN0000003755
TRFP	TRCN0000007285	TRCN0000007286
VEGF	TRCN0000003343	TRCN0000003345
YTHDF1	TRCN0000062769	TRCN0000062770

Supplementary Table 2. Primer index

Gene	Forward primer sequence (5'-3')	Reverse primer sequence (5'-3')
ABHD11	TTCAACTCCATCGCCAAGAT	CACCGTGGTTACGAGCATC
ADORA2B	TCTGTGTCCCGCTCAGGT	GATGCCAAAGGCAAGGAC
AURKB	ATTGCTGACTTCGGCTGGT	GTCCAGGGTGCCACACAT
BIRC5	GCCCAGTGTTTCTTCTGCTT	CCGGACGAATGCTTTTTATG
CENPM	AACACGGCCACCATCTTG	GGGACTTTGCCAAGTGGAC
CHAF1A	GGAGAGGAGAGACGAGCAGA	CTTGCTCCCGTTCACATTG
CHML	TTATCTCCCACCAGGTTCCTC	TTCTCTTATTTCTTCTTTGAAGGTGAT
E2F1	TCCAAGAACCACATCCAGTG	CTGGGTCAACCCCTCAAG
EZH2	TGGTCTCCCCTACAGCAGAA	TCATCTCCCATATAAGGAATGTTATG
FEN1	ACCCCGAACCAAGCTTTAG	GGGCCACATCAGCAATTAGT
FOXM1	ACTTTAAGCACATTGCCAAGC	CGTGCAGGGAAAGGTTGT
H2AFZ	CACCGTGGGTCCGATTAG	GTCCTTTCCAGCCTTACCG
IGFBP3	AACGCTAGTGCCGTCAGC	CGGTCTTCCTCCGACTCAC
IL6	GATGAGTACAAAAGTCCTGATCCA	CTGCAGCCACTGGTTCTGT
MCM10	CATGAAGCCCAAGGATGG	GACCTTCTGAGGATGATCGATAG
MCM2	GCCAAGATGTACAGTGACCTGA	GATGTGCCGCACCGTAAT
MTDH	CTGTCCGAGAAGCCCAAA	TGGCTGCTTTGCTGTTACAC
MYC	GCTGCTTAGACGCTGGATTT	TAACGTTGAGGGGCATCG
PAICS	TTTTCAGTTATTACAGGAAGCAGGT	TGAAAGCTGTCTCCCCACAT
PHLDA1	TCTGCACAAAAACTGGTGAGAC	ACTGCTCAGCCTGCCATC
PPP2R3A	CAGACTCCAGAGGTGATCAAGA	CGGGGACTACTTGGAGAGGT
PTGES	ACGCTGCTGGTCATCAAGA	TCTTCCGCAGCCTCACTT
PTP4A1	GGCCACAATCTTCAATGAGTAA	TGCTGTGCCTGGCAGTAA
RRP1	GCAAGGCTGGGAAGAAAGA	GGGTGCAGGATCTCAGTCAT
SCD	CCTAGAAGCTGAGAAACTGGTGA	ACATCATCAGCAAGCCAGGT
SEC14L1	AGGGGCTGAGTGGTGATG	GTAGTCGGCATCTAGTTTGTCGT
SFN	CAGAGTCCGGCATTGGTC	GCTCTGGGGACACACAGG
SH3GL1	AGGAGGTGGCAGAAACCAG	TGACTCACCTGCTCGATGTC
SMTN	GAACCGACGGAGCTAGGG	CAGATCTGCTGTGACCTCCA
TJAP1	AGAGCTGCCGACAAACAGAC	AGTCATTCTGGGAGGTGACG
TP53	TTCACCCTTCAGATCCGTGG	AGTTCCAAGGCCTCATTCAGC
TRFP	GGAACCCTGCGTTTCTACTG	ACAGGCATCTGGGACACAC
VEGF	CAGACTCGCGTTGCAAGA	GAGAGATCTGGTTCCCGAAA
YTHDF1	CGACGACTTTGCTCACTACG	TTCGACTCTGCCGTTCCTT

Supplementary Table 3. Breast cancer dataset index for Figure 1, Figure 2 and Supplementary Figure 4

# Grade I	# Grade III	Dataset name	PubMed ID	Data source
14	65	Chin 2006	17157792	https://caarraydb.nci.nih.gov/caarray/public ExperimentDetailAction.do?expId= 1015897589973250
30	83	Desmedt 2007	17545524	GEO GSE7390
68	55	Ivshina 2006	17079448	GEO GSE4922
23	64	Li 2010	20098429	GEO GSE19615
14	24	Loi 2008	18498629	GEO GSE9195
28	61	Pawitan 2005	16280042	GEO GSE1456
45	125	Sabatier 2010	20490655	GEO GSE21653
29	35	Schmidt 2008	18593943	GEO GSE11121
64	55	Sotiriou 2006	16478745	GEO GSE2990
75	119	Van DeVijver 2002	12490681	http://www.rii.com/publications/2002/nejm.htm http://microarray-pubs.stanford.edu/wound_NKI/ Clinical_Data_Supplement.xls
